# The role of substantia nigra sonography in the differentiation of Parkinson’s disease and multiple system atrophy

**DOI:** 10.1186/s40035-018-0121-0

**Published:** 2018-07-23

**Authors:** Hai-Yan Zhou, Pei Huang, Qian Sun, Juan-Juan Du, Shi-Shuang Cui, Yun-Yun Hu, Wei-Wei Zhan, Ying Wang, Qin Xiao, Jun Liu, Yu-Yan Tan, Sheng-Di Chen

**Affiliations:** 1 Department of Neurology & Institute of Neurology, Ruijin Hospital affiliated to Shanghai Jiao Tong University School of Medicine, No.197 Ruijin 2nd Road, Shanghai, 200025 China; 2 Department of Ultrasonography, Ruijin Hospital affiliated to Shanghai Jiao Tong University School of Medicine, Shanghai, 200025 China

**Keywords:** Parkinson’s disease, Multiple system atrophy, Atypical parkinsonian disorders, Transcranial sonography, Substantia nigra, Disease duration

## Abstract

**Background:**

The differential diagnosis of Parkinson’s disease (PD) and multiple system atrophy (MSA) remains a challenge, especially in the early stage. Here, we assessed the value of transcranial sonography (TCS) to discriminate non-tremor dominant (non-TD) PD from MSA with predominant parkinsonism (MSA-P).

**Methods:**

Eighty-six MSA-P patients and 147 age and gender-matched non-TD PD patients who had appropriate temporal acoustic bone windows were included in this study. All the patients were followed up for at least 2 years to confirm the initial diagnosis. Patients with at least one substantia nigra (SN) echogenic size ≥18 mm^2^ were classified as hyperechogenic, those with at least one SN echogenic size ≥25 mm^2^ was defined as markedly hyperechogenic.

**Results:**

The frequency of SN hyperechogenicity in non-TD PD patients was significantly higher than that in MSA-P patients (74.1% vs. 38.4%, *p* <  0.001). SN hyperechogenicity discriminated non-TD PD from MSA-P with sensitivity of 74.1%, specificity of 61.6%, and positive predictive value of 76.8%. If marked SN hyperechogenicity was used as the cutoff value (≥ 25 mm^2^), the sensitivity decreased to 46.3%, but the specificity and positive predictive value increased to 80.2 and 80.0%. Additionally, in those patients with SN hyperechogenicity, positive correlation between SN hyperechogenicity area and disease duration was found in non-TD PD rather than in MSA-P patients. In this context, among early-stage patients with disease duration ≤3 years, the sensitivity, specificity and positive predictive value of SN hyperechogenicity further declined to 69.8%, 52.2%, and 66.7%, respectively.

**Conclusions:**

TCS could help discriminate non-TD PD from MSA-P in a certain extent, but the limitation was also obvious with relatively low specificity, especially in the early stage.

## Background

The diagnosis of Parkinson’s disease (PD) mainly depends on the clinical manifestations [1], which results in the accuracy of diagnosis affected by the physicians’ experiences in a great extent. Although as high as 90% accurate diagnosis of PD could be reached in the movement disorders clinics [2, 3], it was already the best scenario. A recent clinical pathology study demonstrated that in the early stage of the disease, only 50% parkinsonian patients received an accurate diagnosis of PD [4]. The most diagnostic confusing diseases are atypical parkinsonian disorders (APD), including progressive supranuclear palsy (PSP), multiple system atrophy (MSA), corticobasal ganglionic degeneration (CBD) and dementia with Lewy bodies (DLB). Those APD share some clinical features with PD, but progress aggressively and have much poorer prognosis. The misdiagnosis not only posed psychosocial and economic burden to patients and their families, but also set up obstacles to the neuroprotective or modified therapies for both PD and APD. Therefore, it is currently eager to find a battery of tools to help diagnosis and differential diagnosis. Transcranial sonography (TCS) is a non-invasive, convenient and economic tool. Recent evidence has shown that it not only helps in the diagnosis of PD, but also helps to differentiate it from APD [5, 6]. However, most of evidence came from Caucasians [6]. Moreover, in most previous differentiation studies not only the number of APD recruited was relatively small, but those distinct entities were pooled into one single APD group for comparison [6]. Among those APD, MSA with predominant parkinsonism (MSA-P), a subtype of MSA, presents most similar clinical features with PD and could be most easily confused with non-tremor dominant (non-TD) PD, especially in the early stage [7, 8]. In our study, we collected 86 clinical probable MSA-P and 147 age and gender matched non-TD PD patients who were clinically diagnosed, and explored if TCS could help the differential diagnosis.

## Methods

This cross-sectional study was performed at the Movement Disorder Clinic, Department of Neurology, Ruijin Hospital affiliated to Shanghai Jiao Tong University School of Medicine from April 2014 to September 2017.

### Subjects

Two hundred and thirty-three Chinese patients with parkinsonism were included, 86 MSA-P and 147 age and gender-matched non-TD PD. All the PD patients met the Movement Disorder Society Clinical Diagnostic Criteria for Parkinson’s disease [9]. Among them, 79 (53.7%) patients met clinically established PD and 68 (46.3%) patients met clinically probable PD at the beginning of recruitment. Moreover, they all met the classification criterion of non-TD PD [10]. Briefly, an average global tremor score was calculated as the mean of 9 items. A mean score for the complex of postural instability and gait difficulty (PIGD) was calculated as the mean of 5 items. The non-TD group was defined as patients with a ratio of mean tremor score/mean PIGD score less than 1.5. Also, the established criterion was used for the diagnosis of probable MSA-P [8]. Cases demonstrated identifiable possible causes of other secondary parkinsonism were excluded. All the patients had appropriate temporal acoustic bone windows and were followed for at least 2 years to confirm the initial diagnosis. During the period of follow-up, no clinical diagnosis was changed. At the end of follow-up, 119 (81.0%) patients met clinically established PD and 28 (19.0%) patients met clinically probable PD. We further analyzed a subgroup of early-stage non-TD PD and MSA-P patients, which was defined as the patients with 3 years or less disease duration. For clinical assessment, disease severity was rated using the Hoehn and Yahr (H-Y) stage.

### Transcranial sonography

The sonographic examinations were done as previously described [11]. Briefly, it was performed by one experienced sonographer blinded to the clinical data of all patients. TCS was performed by a 2.5 MHz sonographic device (MyLab90, ESAOTE, Italy) with 16 cm depth and 45 dB dynamic range. Initially, the butterfly shaped hypoechogenic midbrain was identified. Then, the one with the hyperechogenic signal at the anatomical region of SN in its maximum extent was stored. Bilateral SN echogenic areas were then manually encircled and measured. Reproducibility of the SN sonographic measurement had been previously validated by two independent investigators [11]. The larger value of bilateral SN echogenicity was defined as SN_L_. Those with SN_L_ less than 18 mm^2^ were identified as normal; the others with at least one SN echogenic size ≥18 mm^2^ were classified as hyperechogenic as previously described [11]. Besides, to keep consistent with the literatures [5, 12–15], in this study SN_L_ ≥ 25 mm^2^ was defined as markedly hyperechogenic, and sizes between 18 and 25 mm^2^ as moderately hyperechogenic. For intergroup comparisons, the SN_L_ was used.

### Statistical analyses

All analyses were performed using SPSS 18.0. Continuous variables were given as means (± standard deviation, SD). Categorical variables were summarized by counts of patients and percentages. All variables were tested for normality by Kolmogorov-Smirnov. Two-sample T test or Chi-square test was applied for the comparison of clinical data between non-TD PD and MSA-P. For the comparison of the frequency of different echogenic intensity between groups, Chi-square test was used. The sensitivity, specificity, positive predictive value (PPV) and negative predictive value (NPV) of TCS were determined for SN hyperechogenicity as a marker for the differential diagnosis of non-TD PD from MSA-P. Correlation analyses between the variables and SN_L_ were performed by Spearman correlation coefficients. Adjusted *p* values were calculated by controlling other confounding factors. The significant level was set at α = 0.05.

## Results

### Demographics

Table 1 presents the demographic data of non-TD PD and MSA-P. Group MSA-P consisted of 57 men and 29 women with clinically probable MSA. Their age was 62.3 ± 7.5 years, onset age 58.7 ± 7.6 years, disease duration 3.6 ± 2.1 years, and H-Y stage 3.5 ± 1.0. Group non-TD PD consisted of 104 men and 43 women. Their age was 62.2 ± 8.4 years, onset age 56.8 ± 8.9 years, disease duration 5.4 ± 4.4 years, and H-Y stage 2.0 ± 0.8. The age and gender were matched between those two groups (*p* = 0.912 and *p* = 0.557, respectively). No difference was found in their onset age (*p* = 0.096). However, MSA-P patients had shorter disease duration (*p* <  0.001) and higher H-Y stage (*p* <  0.001).

**Table 1:** Demographic data and substantia nigra echogenicity of patients

	non-TD PD (*n* = 147)	MSA-P (*n* = 86)	P value
Age (y)	62.2 (8.4)	62.3 (7.5)	0.912
Gender (M/F)	104/43	57/29	0.557
Onset age (y)	56.8 (8.9)	58.7 (7.6)	0.096
Disease duration (y)	5.4 (4.4)	3.6 (2.1)	< 0.001^*^
H-Y stage	2.0 (0.8)	3.5 (1.0)	< 0.001^*^
SN_L_ (mm^2^)	24.7 (12.8)	12.2 (13.6)	< 0.001^*^

*Non-TD PD* non-tremor dominant Parkinson’s disease, *MSA-P* multiple system atrophy with predominant parkinsonism, *H-Y* Hoehn and Yahr, *SN*_*L*_ the larger value of bilateral SN hyperechogenicity. Two-sample T test or Chi-square test was applied for the comparisons. ^*^P values below 0.05 were considered significant

### SN echogenicity of non-TD PD and MSA-P

**Figure 1:**
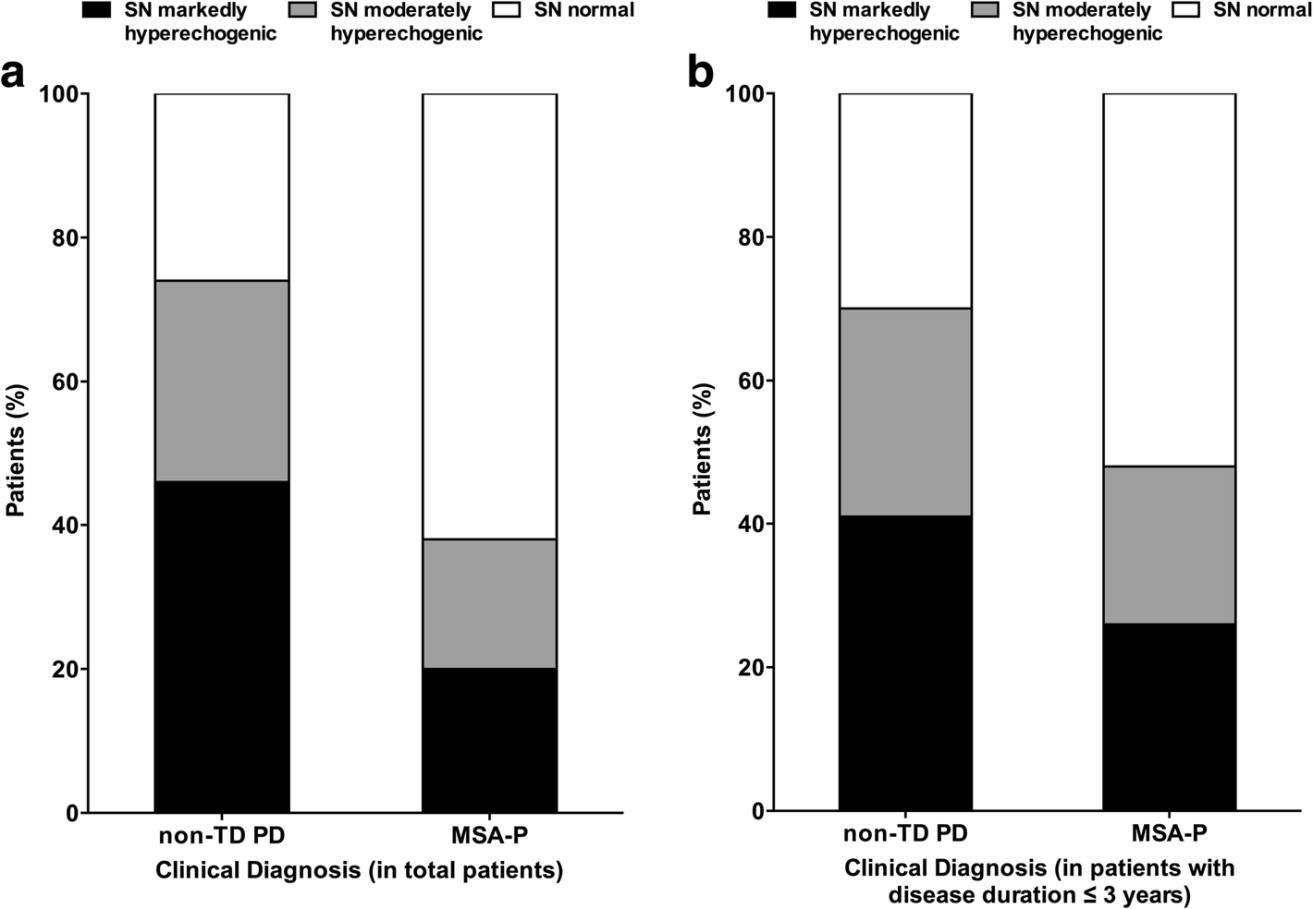
Percentage of patients with different ultrasound findings in the two groups. **a** showed the percentage of cases with different sonographic findings in total patients. **b** showed the percentage of cases with different sonographic findings in the subgroup of patients with disease duration ≤3 years. Black: percentage of patients with a marked substantia nigra (SN) hyperechogenicity; grey: percentage of patients who exhibited a moderate increase in SN echogenicity; and white: percentage of patients with a normal SN echogenicity; non-TD PD, non-tremor dominant Parkinson’s disease; MSA-P, parkinsonian variant of multiple system atrophy.

The mean SN_L_ of non-TD PD was 24.7 ± 12.8 mm^2^, larger than that of MSA-P patients, which was 12.2 ± 13.6 mm^2^ (*p* <  0.001). In non-TD PD group, 109 (74.1%) of 147 patients exhibited SN hyperechogenicity. Forty-one (27.9%) were classified as moderate, and 68 (46.3%) as marked. Thirty-eight (25.9%) of 147 non-TD PD patients had normal SN echogenicity. On the contrary, in the MSA-P group, only 33 (38.4%) of 86 patients exhibited SN hyperechogenicity, and the frequency was lower than that in non-TD PD (*P* <  0.001). Among them, 16 (18.6%) patients were classified as moderate, and 17 (19.8%) as marked. Fifty-three (61.6%) patients exhibited normal SN echogenicity (Fig. 1a**)**.

In the subgroup of patients with disease duration ≤3 years (*n* = 109), the mean SN_L_ of non-TD PD (*n* = 63) was 23.9 ± 12.0 mm^2^, also larger than that of MSA-P patients (*n* = 46), which was 15.2 ± 14.9 mm^2^ (*p* = 0.002). In non-TD PD group, 44 (69.8%) of 63 patients exhibited SN hyperechogenicity. Eighteen (28.6%) of 63 patients were classified as moderate, and 26 (41.3%) patients as marked. Nineteen (30.2%) of 63 non-TD PD patients had normal SN echogenicity. On the contrary, in the MSA-P group, 24 (52.2%) of 46 patients exhibited normal SN echogenicity and 22 (47.8%) patients exhibited SN hyperechogenicity. Among them, 10 (21.7%) patients were classified as moderate, and 12 (26.1%) patients as marked (Fig. 1b**)**.

### SN hyperechogenicity differentiating non-TD PD from MSA-P

Table 2 summarizes SN sonographic findings discriminating non-TD PD from MSA-P. Of all the 233 patients, SN echogenicity correctly diagnosed 162 (69.5%) cases as non-TD PD (*n* = 109) or MSA-P (*n* = 53). For the clinical diagnosis of non-TD PD, sensitivity, specificity and PPV of SN hyperechogenicity were 74.1%, 61.6% and 76.8%; while for being diagnosed as MSA-P, normal SN echogenicity had a NPV of 58.2%. For marked SN hyperechogenicity, sensitivity, specificity and PPV for the diagnosis of non-TD PD were 46.3%, 80.2% and 80.0%, respectively. Conversely, normal SN echogenicity indicated MSA-P with 46.6% NPV.

**Table 2:** Substantia nigra sonography findings indicating non-TD PD rather than MSA-P

	Total patients		Patients with disease duration ≤3 years	
	SN_L_ ≥ 18 mm^2^	SN_L_ ≥ 25 mm^2^	SN_L_ ≥ 18 mm^2^	SN_L_ ≥ 25 mm^2^
Sensitivity, %	74.1	46.3	69.8	41.3
Specificity, %	61.6	80.2	52.2	73.9
PPV, %	76.8	80.0	66.7	68.4
NPV, %	58.2	46.6	55.8	47.9

*Non-TD PD* non-tremor dominant Parkinson’s disease, *MSA-P* multiple system atrophy with predominant parkinsonism, *SN*_*L*_ the larger value of bilateral SN hyperechogenicity, *PPV* positive predictive value, *NPV* negative predictive value

In the subgroup of patients with disease duration ≤3 years (*n* = 109), SN echogenicity correctly diagnosed 68 (62.4%) cases as non-TD PD (*n* = 44) or MSA-P (*n* = 24). SN hyperechogenicity had 66.7% PPV for the clinical diagnosis of non-TD PD and normal SN echogenicity had 55.8% NPV for being diagnosed as MSA-P. Sensitivity and specificity of SN hyperechogenicity for detecting non-TD PD patients decreased to 69.8% and 52.2%. For marked SN hyperechogenicity, sensitivity, specificity, and PPV for non-TD PD were 41.3%, 73.9% and 68.4%. Conversely, normal SN echogenicity indicated MSA-P with 47.9% NPV.

### Correlation of SN_L_ with disease duration in the patients with SN hyperechogenicity

**Figure 2:**
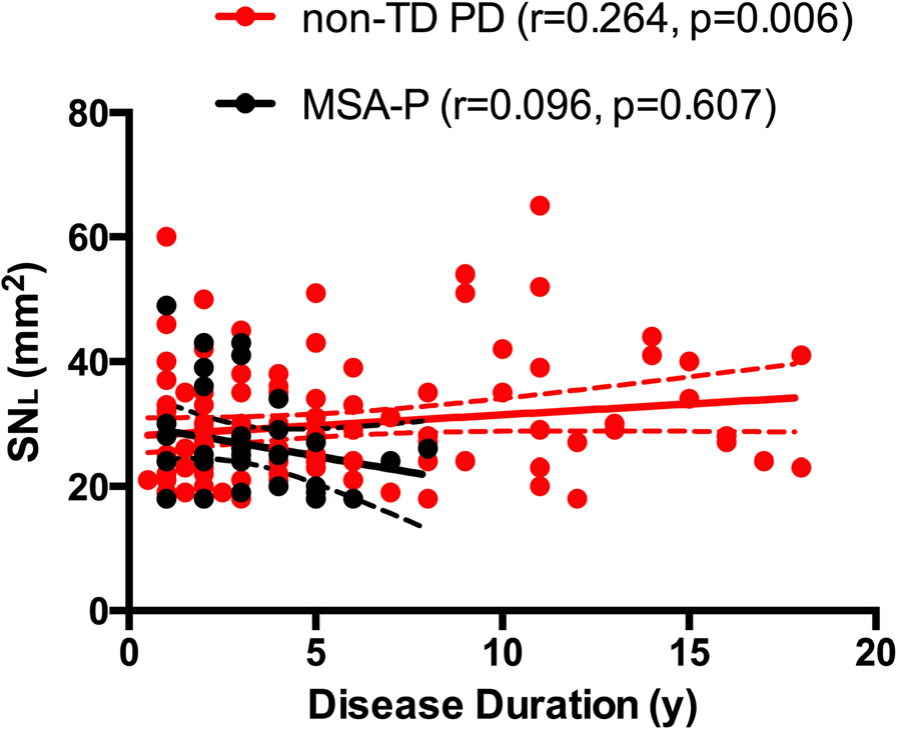
Correlation of SN_L_ area with disease duration in patients with SN hyperechogenicity

Correlation analysis of SN_L_ with disease duration was performed in patients with SN hyperechogenicity. The results indicated that SN_L_ did not correlated with disease duration either in MSA-P (*r* = − 0.114, *p* = 0.526) or in non-TD PD patients (*r* = 0.137, *p* = 0.156). However, after controlling onset age and H-Y stage as confounding factors, significant correlation was found only in non-TD PD patients (*r* = 0.264, *p* = 0.006) rather than in MSA-P patients (*r* = − 0.096, *p* = 0.607) (Fig. 2).

## Discussion

Consistent with previous results [12, 15, 16], our present study demonstrated that more percentage of SN hyperechogenicity was detected in non-TD PD patients than in MSA-P patients. However, the sensitivity for the non-TD PD patients in our cohort was only 74.1%, which was lower than most of previous studies performed by other researchers [5, 6, 12–22]. The majority of earlier studies were conducted in Caucasians and the sensitivity was almost more than 90%, even reaching up to 100% [5, 6, 12–19]. Only a few studies were performed in the Asian patients, and the sensitivity varied widely (50%~ 80%) [11, 20–28]. Ethnic differences might not be completely excluded between Caucasians and Asians, but the spectrum bias should be the more reasonable cause. It has been well known that PD is a heterogeneous disorder, and it can be divided into various phenotypes based on the onset age or prominent clinical presentations, such as early or late onset PD, TD PD or non-TD PD [29–31]. Different patterns of SN echogenicity have been shown to be associated with different clinical subtypes [32] and different onset ages [22]. Also, gender and disease duration could affect the SN echogenic pattern [11, 20, 22, 33]. In view of above-mentioned various factors, it was not surprising that varied sensitivity was found in our own studies. Lower sensitivity in our previous studies with a larger number of PD patients could also be due to different patient’s enrollment standard [11]. In the current study, PD patients were limited to the non-TD patients, and their ages and genders were strictly matched with that of MSA-P. Finally, the possibility of misdiagnosis could not be absolutely ruled out since a fairly part of non-TD PD patients were at their early disease stages. However, similar with a recent study conducted in Japanese patients where the sensitivity of SN hyperechogenicity was around 50% [23], all the patients in our cohort were followed up for at least 2 years to confirm the diagnosis. In supporting for the minimum possibility of misdiagnosis, the differing characteristics of non-TD PD and MSA-P in our study were obvious, that is, MSA-P patients had higher H-Y stages with shorter disease duration, which suggesting more aggressive progression.

In contrast, the sensitivity of SN hyperechogenicity for MSA-P was 38.4%. Even only the marked SN hyperechogenicity was considered, the sensitivity was up to 19.8%, which was still higher than most of previous reports [5, 6, 12, 15, 16, 18, 19, 21, 23]. The majority of sensitivity of SN hyperechogenicity for MSA was reported as about 10%, except for two studies, which were 25 and 50%, respectively [12, 27]. Just as occurred in PD, the most probable reason for the discrepancy between earlier studies and ours could be the spectrum bias. In the previous studies, the number of MSA patients enrolled was relatively small and ranged from 8 to 32 [5, 6]. A small sample size could not only affect statistical power, but also influence the statistical results even with a few cases of misdiagnosis. Moreover, there are two variants of MSA, which are MSA with predominant cerebellar ataxia (MSA-C) and MSA-P. No evidence could make sure that their SN hyperechogenicity pattern should be the same. Therefore, to minimum the bias mentioned above, the MSA patients selected in our study were all MSA-P variants and were followed up for at least two years to confirm the diagnosis in the same movement disorder clinic. In addition, the sample size of MSA-P in the present study reached up to 86, which was the largest one so far.

For overall patients, the sensitivity of SN hyperechogenicity for PD was 74.1%, and the specificity was 61.6%. If SN hyperechogenicity was marked, the specificity increased to 80.2%, but the sensitivity decreased to 46.3%. With either cutoff value, the positive predictive value for the diagnosis of non-TD PD was around 80%. However, among the patients with equal to or less than 3 years disease duration, the sensitivity and specificity further declined to 69.8% and 52.2% respectively, so did PPV decrease to 66.7%. The exact reason was not clear, and the possibility of misdiagnosis could not be definitely excluded since a fairly part of patients were at their early stages. However, it might be explained by the fact that there were different patterns between non-TD PD and MSA-P with regard to the correlation of SN hyperechogenicity with disease duration. In non-TD PD, it seems that with disease course prolonged, the area of SN hyperechogenicity became larger, which was discrepant with recent studies indicating that SN hyperechogenicity was stable during disease progression [17, 32, 34, 35]. Although the correlation between SN_L_ and disease duration was weak, several investigations [20, 22, 33] and our previous study [36] did support this positive correlation. In contrast, a tendency of reversed correlation was found in MSA-P patients, although without statistical significance. Therefore, it could be expected that within shorter disease duration, non-TD PD patients had smaller SN echogenicity, which could lead to lower percentage of SN hyperechogenicity, whereas MSA-P patients might have larger SN echogenicity, which could lead to higher percentage of SN hyperechogenicity. Additionally, this different correlation of SN hyperechogenicity with disease duration might also support the hypothesis about the distinct underlying mechanisms in the pattern of SN echogenicity of PD and MSA. So far, the origin of SN hyperechogenecity was not completely elucidated, nonchelated forms of iron, protein bound iron and microglia activation were all supposed to be involved in the reflection of SN hyperechogenecity signals [33, 37, 38]. Tissue iron has been shown to be elevated in both PD and MSA-P [39], but the detailed iron metabolism and gliosis might be different in PD and MSA [33, 37–39].

Limitations of this study need to be mentioned. Indeed, we could not promise absolutely accurate diagnosis of PD and MSA, since no post-mortem verification was achieved. This also was the problem for the differentiation between MSA and other APD, such as CBD and DLB. SN hyperechogenicity has been reported frequent in CBD and DLB [40, 41], therefore misdiagnosis of those disorders could also contribute to higher sensitivity of SN hyperechogenicity in MSA-P. Additionally, although non-TD PD was considered as the phenotype that was most difficult to discriminate from MSA-P, one should note that non-TD and TD PD subtypes do not necessarily represent different subtypes of PD. TD subtype was much more likely to shift to non-TD subtype during the disease progression [42].

## Conclusions

In summary, our present study demonstrated that SN hyperechognicity could help to discriminate non-TD PD from MSA-P; however, both sensitivity and specificity were not satisfactory, particularly in the earlier stage. Combination with other auxiliary tests seemed a necessary way to help differentiation of these two entities.

## Abbreviations

APD: Atypical parkinsonian disorders
CBD: Corticobasal ganglionic degeneration
DLB: Dementia with Lewy bodies
H-Y: Hoehn and Yahr
MSA: Multiple system atrophy
MSA-C: MSA with predominant cerebellar ataxia
MSA-P: MSA with predominant parkinsonism
non-TD PD: Non-tremor dominant PD
NPV: Negative predictive value
PD: Parkinson’s disease
PIGD: Postural instability and gait difficulty
PPV: Positive predictive value
PSP: Progressive supranuclear palsy
SN: Substantia nigra
TCS: Transcranial sonography
